# Selective orthotic constraint of lower limb movement during walking reveals new insights into neuromuscular adaptation

**DOI:** 10.3389/fresc.2024.1354115

**Published:** 2024-06-27

**Authors:** Christopher F. Hovorka, Géza F. Kogler, Young-Hui Chang, Robert J. Gregor

**Affiliations:** ^1^ Department of Rehabilitation Medicine, Center for the Intrepid, Brooke Army Medical Center, San Antonio, TX, United States; ^2^ Defense Health Agency, Falls Church, VA, United States; ^3^ Oak Ridge Institute for Science and Education, Oak Ridge, TN, United States; ^4^ Clinical Biomechanics Laboratory, Kennesaw State University, Kennesaw, GA, United States; ^5^ School of Biological Sciences, Georgia Institute of Technology, Atlanta, GA, United States; ^6^ School of Integrated Health Sciences, University of Nevada Las Vegas, Las Vegas, NV, United States

**Keywords:** ankle foot orthosis, neuromuscular, soleus, tibialis anterior, footwear

## Abstract

**Introduction:**

A concern expressed by the clinical community is that the constraint of motion provided by an ankle foot orthosis (AFO) may lead the user to become dependent on its stiffness, leading to learned non-use. To examine this, we hypothesized that using an experimental AFO-footwear combination (exAFO-FC) that constrains ankle motion during walking would result in reduced soleus and tibialis anterior EMG compared to free (exAFO-FC) and control (no AFO, footwear only) conditions.

**Method:**

A total of 14 healthy subjects walked at their preferred speed (1.34 ± 0.09 m·s-1) for 15 min, in three conditions, namely, control, free, and stop.

**Results:**

During the stance phase of walking in the stop condition, ipsilateral soleus integrated EMG (iEMG) declined linearly, culminating in a 32.1% reduction compared to the control condition in the final 5 min interval of the protocol. In contrast, ipsilateral tibialis anterior iEMG declined in a variable fashion culminating in an 11.2% reduction compared to control in the final 5 min interval. During the swing phase, the tibialis anterior iEMG increased by 6.6% compared to the control condition during the final 5 min interval. The contralateral soleus and tibialis anterior exhibited increased iEMG in the stop condition.

**Discussion:**

An AFO-FC functions as a biomechanical motion control device that influences the neural control system and alters the output of muscles experiencing constraints of motion.

## 1 Introduction

Ankle foot orthoses (AFOs) are one of the most commonly prescribed orthoses (1), designed to provide stability to a user during standing while also optimizing gait when functional deficits (e.g., loss of dorsiflexion and loss of plantarflexion) are present. An AFO combined with footwear (AFO-FC) controls joint motion at the ankle and the knee in a prescribed manner to improve walking performance by minimizing or eliminating undesirable compensatory pathological gait patterns (2–16). Current clinical practice is informed by basic and intuitive mechanical solutions in movement control (e.g., assist, resist, and stop) (17) that have relatively predictable outcomes in gait mechanics. Unfortunately, this limited clinical perspective with a focus on joint position and mechanical control overlooks the neuromuscular and sensorimotor response mechanisms associated with ankle joint control. A small number of studies using passive AFOs (18) and footwear have examined subjects’ neuromuscular output of muscles that act on the ankle by using electromyography (EMG) or other measures to describe the magnitude of muscle response (19–32). The goal of this investigation was to improve our understanding of neuromuscular output during the early adaptation period to the constraint of ankle joint motion by an AFO-FC. Our strategy was to use EMG to monitor muscle activation output based upon the premise that EMG records electrical signals in the muscle action potential and hence provides a window of nervous system control of muscle activation during movement. Accordingly, we sought to better understand the consequential neuromuscular considerations between constrained and unconstrained ankle motion using an AFO combined with footwear, by collecting EMG activity of lower limb muscles during treadmill walking in healthy subjects.

A concern expressed by the clinical community is that the constraint of motion provided by an orthosis may lead the user to be dependent on the stiffness and stability provided by the orthosis to the lower limb during standing and walking. This continued dependence over a prolonged period of AFO and footwear use will lead to learned non-use (33) and muscle atrophy (22, 31). Of these studies, the largest cohorts of subjects that used AFO-FCs were hemiparetic stroke survivors during the subacute phase of recovery. Reported results are conflicting. Murayama and Yamamoto (22) showed that subjects' use of an AFO-FC elicited differences in the EMG magnitude of the tibialis anterior muscle over 16 weeks, whereas Nikamp et al. (23) reported no difference in the magnitude of tibialis anterior activity after 26 weeks of AFO-FC use. Geboers et al. examined patients with lower limb peripheral neuropathy and reported a modest 6% decline in the EMG magnitude of the tibialis anterior muscle after 6 weeks of use of an AFO compared to a 20% decline in the EMG of the tibialis anterior muscle in patients that did not use an AFO (19). The optimal prescription recommendation for any individual type of AFO-FC design, including the dose of use (e.g., frequency, intensity, and duration), is critical to any schedule of neuromotor rehabilitation. To begin addressing these concerns, this study sought to characterize and quantify a relationship between the constraint of joint motion and neuromuscular output using EMG and motion capture. We hypothesized that the use of an experimental AFO-footwear combination (exAFO-FC) that constrained ankle motion during walking would reduce the magnitude of tibialis anterior and soleus muscle EMG compared to a free (exAFO-FC) condition and a control (no AFO, footwear only) condition.

## 2 Materials and methods

### 2.1 Subjects

A total of 14 healthy subjects with right leg dominance [eight females; six males; mean (standard deviation) ages, 21.04 (0.89) years; height, 171.19 (4.11) cm; mass, 65.74 (4.72) kg] gave written informed consent to participate in a protocol approved by the Georgia Institute of Technology Institutional Review Board.

### 2.2 Instrumentation, limb segment modeling, and computation

The study involved a 3D gait lab using six high-speed cameras (Vicon, Oxford, UK; 120 Hz) and 16 retroreflective markers (14 mm diameter) taped to the pelvis and lower limbs of subjects using a method modified by Kadaba et al. (34) to record joint motion. Specific anatomical sites for marker placement were as follows: anterior superior iliac spine, posterior superior iliac spine, thigh segment, knee joint center, shank segment, lateral malleolus, calcaneus, and second metatarsophalangeal joint (34). Because the visibility of markers attached to the skin of subjects’ shank and foot regions was impeded by the AFO, to restore visibility, we attached markers to the exterior of the orthosis at the shank, lateral ankle joint, heel strap at the calcaneus, and forefoot strap at the dorsum of the second metatarsophalangeal joint.

A custom dual belt treadmill with embedded force plates, one under each belt (AMTI, Watertown, MA, USA; 1,080 Hz), was used to collect ground reaction forces, joint moments, and temporospatial parameters (i.e., stance duration, swing duration, and cadence). All data were collected in the Vicon workstation and motion data were processed using the plug-in-gait model to identify and label markers. All data were imported to MATLAB version 7.11.0 (The MathWorks Inc., Natick, MA, USA) for additional processing. Raw force signals were filtered (fourth-order Butterworth low-pass filter with a cutoff frequency of 20 Hz) and analyzed to determine ground reaction components and joint moments during the stance phase and to identify the duration of stance and swing phases. Motion data were filtered (fourth-order Butterworth low-pass filter with a cutoff frequency of 10 Hz) and analyzed to determine the angular motion of the ankle joint. All motion and force data were synchronized and time normalized to 100% of the gait cycle and analyzed using standard inverse dynamic calculations and estimated inertial characteristics based on subject-specific anthropometrics (35). Because the dominant motions of the ankle joint complex occur through the talocrural articulation as plantarflexion and dorsiflexion during gait, analysis of ankle motion was restricted to the sagittal plane (36, 37).

Activation of the tibialis anterior and soleus muscles was sampled on both legs using wireless electromyography (EMG) (Noraxon USA, Scottsdale, AZ, USA; 1,500 Hz) and bipolar Ag/Ag-Cl adhesive electrodes (Danlee Medical Products, Syracuse, NY, USA) incorporating 20 mm inter-electrode spacing were adhered to the skin of subjects. We recorded kinematic, kinetic, and EMG data from each subject during the first 30 s of every minute as they walked on a treadmill at their self-selected speed.

### 2.3 Leg dominance and EMG

To account for any gait variations (38–40) (e.g., dominant vs. non-dominant) that may contribute to muscle adaptation, the dominant leg of each subject was identified by administering three motor tasks (ball kick, step ascent, and standing balance recovery) (41–44). Subjects with right leg dominance were selected for the study. We collected surface EMG of the principal single-joint muscles that provide ankle motion during walking (ipsilateral and contralateral soleus and tibialis anterior) because these muscles are likely to be influenced by the AFO (45) The surface electrode locations were determined by the principal investigator (CH) in accordance with the European standards of surface EMG for non-invasive assessment of muscles (SENIAM) to minimize impedance and maximize EMG signal fidelity (46). A ground electrode was attached to the skin of each subject's left leg at the proximal anteromedial plateau of the tibia. To minimize the risk of motion artifact, pre-amplifiers and EMG cables were wrapped and secured to the subjects' legs and adjusted to allow a typical range of hip, knee, and ankle motion for walking. To ensure fidelity and minimize cross talk, we visually inspected the EMG signals of each participant during manual muscle tests and 5 m overground walking tests prior to treadmill walking, moving electrode placement if needed.

### 2.4 Preferred walking speed

Preferred walking speed was determined by administering three trials of the 10 m walk test for overground walking (47). The mean overground walking speed was then matched to individuals' treadmill speeds by adapting a method described by Amorim et al. (48).

### 2.5 Experimental AFO and footwear

An experimental AFO (exAFO) with integrated footwear (total mass of 1.76 kg) was designed and created to fit the right (dominant side) leg of all subjects. To achieve this, the AFO included sufficient adjustability at the foot and shank to provide an intimate fit and to provide multiple motion control conditions including maximum constraint of ankle motion in a stop condition and free ankle motion in a free condition through an adjustable clamp and low-friction sliding bearing system (3). To minimize the variability of footwear and limb length and to maintain rollover dynamics when the ankle was constrained by the AFO, we integrated a footwear system. The motion control performance of the experimental AFO-footwear combination (exAFO-FC) was validated in quasistatic loading experiments using cadaveric limbs and human subject treadmill walking experiments in an instrumented gait lab (3).

To ensure proper fit of the exAFO-FC and congruency between the anatomical and orthotic ankle joints, subject inclusion criteria specified a range for individual foot length, ankle height, and calf girth. More specifically, key design features were included in the experimental AFO-FC to minimize displacement of subjects' shank and foot. For example, an adjustable heel strap secured the hindfoot, an adjustable dorsal foot strap secured the midfoot, a rigid foot shell secured the forefoot, and a rigid shank shell with an adjustable tibial plate secured the leg. An adjustable linear slide bearing was anchored between the shank and foot shells to maximally constrain ankle motion (clamps secured) or to allow free ankle motion (clamps removed).

Alignment of the exAFO-FC in the stop condition, for all 14 legs, was set at a shank-to-vertical angle 10° incline (i.e., modest ankle dorsiflexion angle) based on the findings reported by Owen (4) to facilitate rollover during stance phase. The experimental AFO-FC was necessary for this study as commercial and custom orthoses with integrated footwear were neither available nor were they validated to meet the rigorous requirements for ankle motion control and stance phase rollover performance for this study.

### 2.6 Experimental protocol

**Figure 1:**
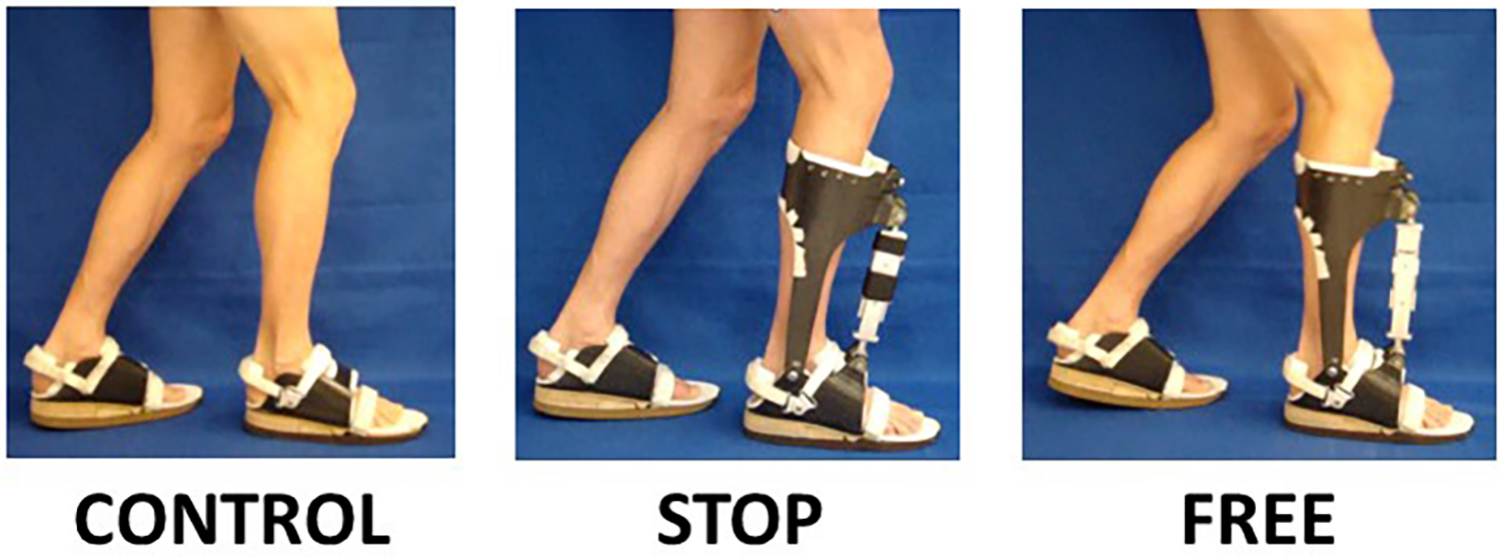
Experimental AFO and footwear conditions. Subjects treadmill walked in the control condition (use of bilateral footwear, no AFO), stop condition (use of contralateral footwear with ipsilateral AFO and integrated footwear in maximal constraint with clamps installed in linear slide bearing), and free condition (use of contralateral footwear with ipsilateral AFO and integrated footwear in no constraint condition with clamps removed from linear slide bearing).

The subjects were tested walking at their preferred speed (1.34 ± 0.09 m·s-1) for 15 min, in three conditions, namely, control (bilateral footwear combination, no AFO), free (use of contralateral footwear with ipsilateral AFO-FC in no constraint condition), and stop (use of contralateral footwear with ipsilateral AFO-FC in maximal constraint) (Figure 1). To wash out the carryover effects between the stop and free conditions, the subjects walked at their preferred speed (1.34 ± 0.09 m·s-1) for 15 min in the control condition. The order of the two exAFO-FC conditions was randomized to minimize the order effects.

### 2.7 Data processing and analysis

All motion and force data in the sagittal plane and all EMG data were synchronized, filtered, and time normalized to 100% of the gait cycle. The mean ground reaction force, moments, and angles, for the stop and free conditions and the 95% confidence interval for the control condition were calculated. The mean ankle range of motion (ROM) in each condition was analyzed using repeated measures ANOVA with Bonferonni *post hoc* comparison.

**Figure 2:**
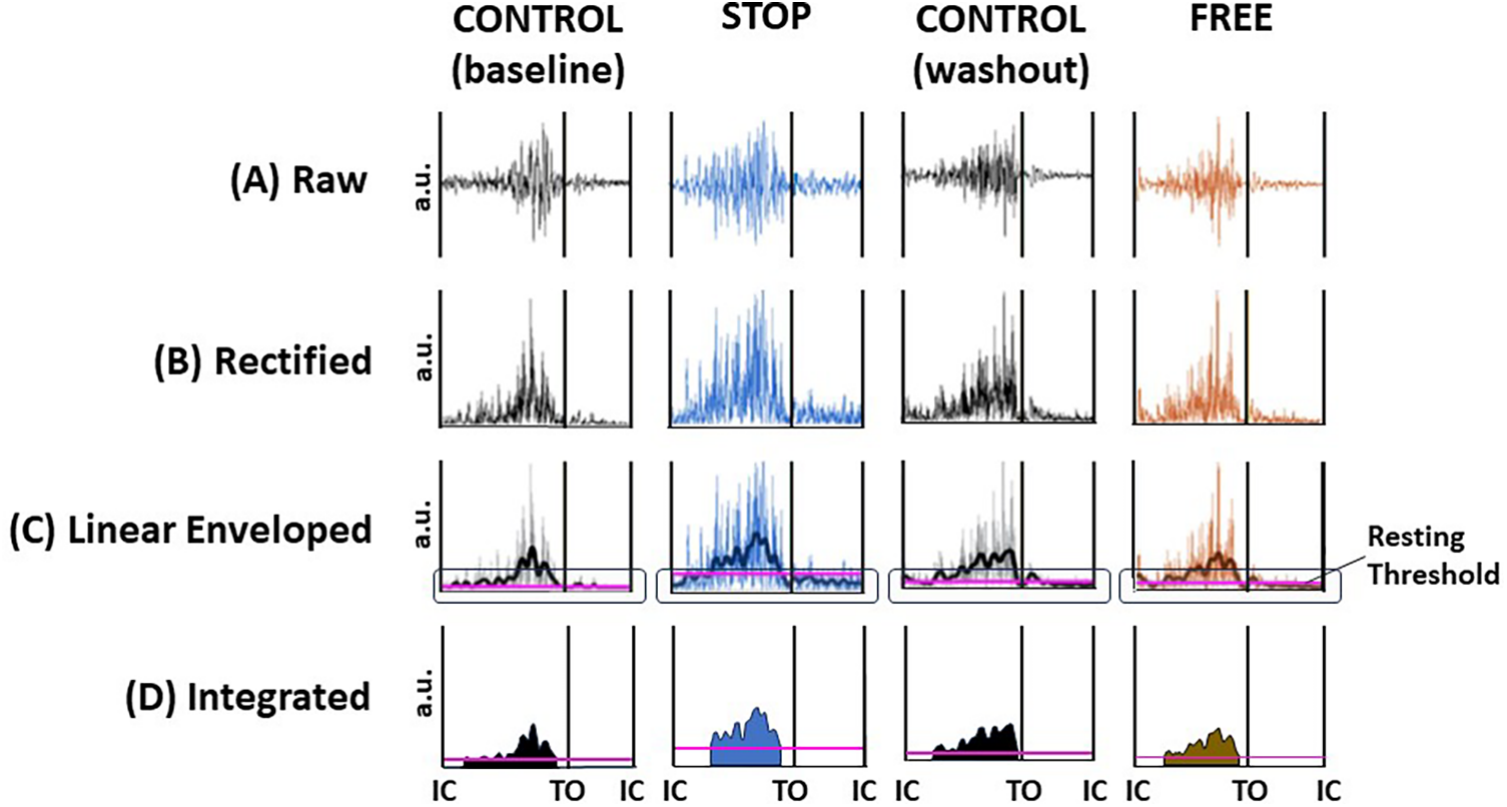
Digital signal processing of EMG data. Exemplar data of a subject's ipsilateral soleus muscle during minute 13, gait cycle 9 in the control (baseline), stop, control (washout), and free conditions. (**A**) raw EMG, (**B**) full wave rectified EMG, (**C**) band-pass and low-pass filtering to render a linear enveloped EMG (jagged line). Resting threshold (horizontal purple line encircled) to identify the onset and termination of each burst activation period. (**D**) Integrated EMG (iEMG) is calculated as the integration of the linear enveloped area of EMG and represents the quantity of the area under the rectified and enveloped EMG and hence the quantity of total activation. Resting threshold (horizontal purple line). The black vertical lines represent initial contact (IC) and toe off (TO) events of the gait cycle; voltage in arbitrary units (au).

Raw EMG data for all subjects' soleus and tibialis anterior muscles were synchronized with force and motion data in the Vicon workstation and were exported offline to MATLAB version R2009a (The MathWorks Inc., Natick, MA, USA) for further processing. The raw EMG data (Figure 2A) were adjusted for voltage offsets, full wave rectified, and band-pass filtered with frequency cutoffs at 10–500 Hz (Figure 2B), followed by the application of a fourth-order Butterworth filter with zero lag at a cutoff frequency of 20 Hz to obtain linear envelopes and rectification to adjust for signal offsets due to enveloping the data (Figure 2C). The cutoff frequency of 20 Hz was selected because it produced smoothed signals that closely represented the shape of each muscle's raw EMG tension curves while still retaining the signals' critical temporal characteristics. A resting threshold was calculated to identify the onset and termination of muscle burst activation (49–52). The area under the rectified and linear enveloped EMG during each burst activation period was calculated as the integrated EMG (iEMG) (Figure 2D). Hence, the iEMG was a representation of the quantity of each muscle's total activation during the burst activation period.

### 2.8 Analysis of soleus and tibialis anterior iEMG during each step

In the first analysis, we quantified and characterized subjects' tibialis anterior and soleus muscle adaptation during each step in each condition (e.g., control, free, and stop). We calculated each subject's integrated EMG during each burst activation period of the tibialis anterior and soleus muscles of ipsilateral and contralateral legs in the control, free, and stop conditions throughout the 15 min walking period. Due to the occasional loss of EMG signal fidelity, the iEMG data were collapsed into seven continuous gait cycle intervals. The baseline reference value was calculated as the mean of the pooled iEMG of both legs (ipsilateral and contralateral) of all subjects in the control condition for the entire 15 min walking period. We calculated the mean iEMG of all subjects for the respective leg (ipsilateral and contralateral) and muscle (tibialis anterior and soleus) in each of the seven gait cycle intervals for each condition (control, free, and stop).

### 2.9 Analysis of the relationship of soleus and tibialis anterior iEMG and ankle ROM during the last 5 min

In the second analysis, we quantified the relationship between subjects' tibialis anterior and soleus muscle and ankle ROM during the final 5 min in each condition (e.g., control, fee, and stop). We selected the final 5 min for comparative analysis. This interval was selected because subjects exhibited the least variability in ankle motion, which is an indication that steady-state gait was achieved. A baseline reference value (mean control) was calculated as the pooled iEMG of both legs (ipsilateral and contralateral) of all subjects (*n* = 14) in the control condition and as the pooled ankle ROM. For all subjects (*n* = 14), we calculated the mean iEMG for each leg (ipsilateral and contralateral), each muscle (tibialis anterior and soleus), and each condition (control, free, and stop). We analyzed the difference in subjects' mean iEMG during each condition (control, free, and stop) using a one-tailed, paired student's *t*-test. We similarly analyzed the difference in the subject's mean ankle ROM. All EMG and muscle activation data were analyzed using SPSS (IBM SPSS Statistics for Windows, version 21.0, Armonk, NY, USA).

## 3 Results

### 3.1 Force, motion, and temporal-spatial outputs during gait

Subjects' use of an experimental AFO-footwear combination elicited a substantial decrease in ipsilateral ankle ROM, to within a mean (standard deviation) of 3.7 (2.1)° in stop, compared to 27.7 (4.2)° in control (*p *= 0.000), and 24.2 (3.6)° in free (*p *= 0.091). There were no differences in ipsilateral ankle moments (*p *> 0.05) and no difference in ankle motion and moments in the three conditions on the contralateral leg (*p *> 0.05). Additionally, there were no differences (*p *> 0.05) in step length, but significant (*p *< 0.05) yet modest differences in stance duration (4%) and swing duration (6%) were elicited by subjects during gait, which suggests a near absence of compensatory movements. The force, motion, and temporospatial outputs reported were during the fourth minute of walking, which was the onset of steady-state gait. Steady-state gait was determined as the onset of minimal variability which began in the fourth minute and remained consistent for the remainder of the 15 min walking period. Additional details regarding these findings are available in a prior published study (3).

### 3.2 EMG of soleus and tibialis anterior muscles during gait

#### 3.2.1 Integrated EMG of soleus and tibialis anterior muscles during continuous stepping

**Figure 3:**
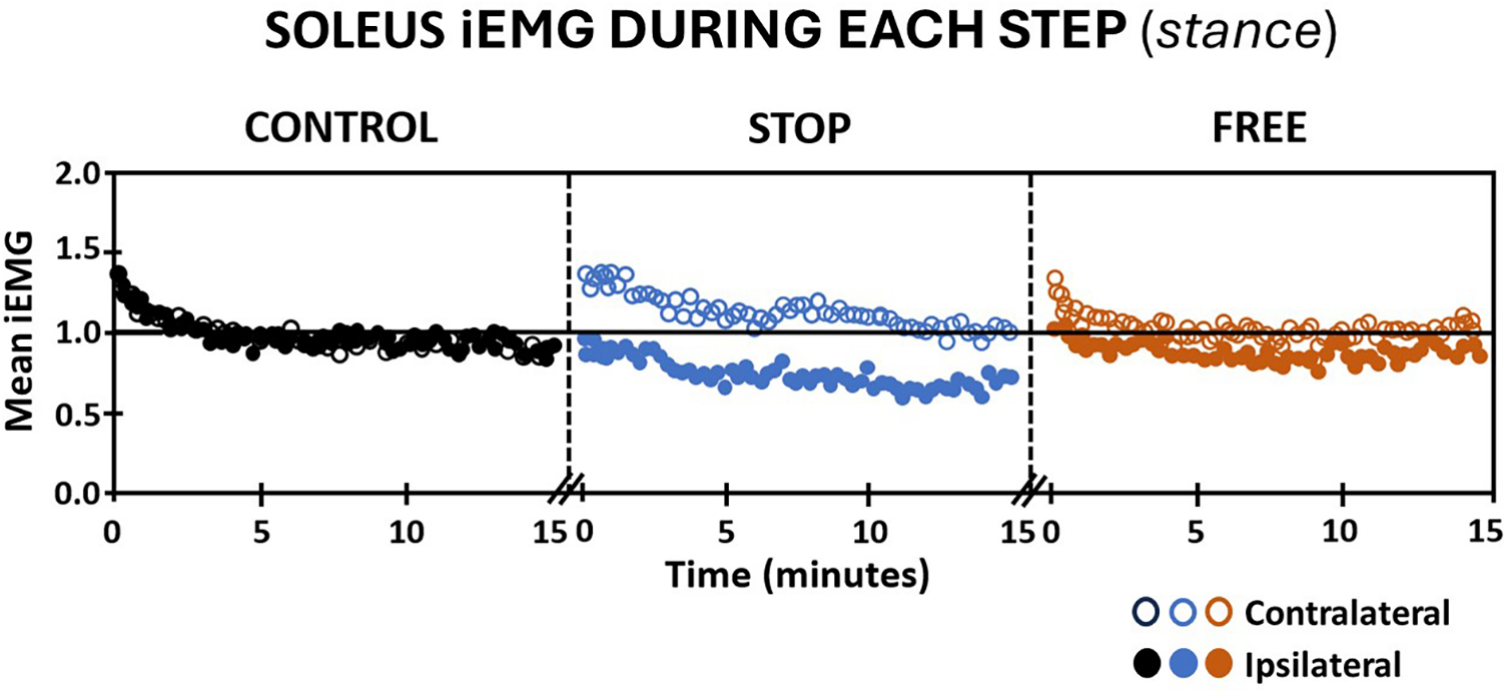
Soleus muscle activation (mean iEMG) during each step (stance phase) in the control (black), stop (blue), and free (brown) conditions during walking for all subjects (*n* = 14). Note the ipsilateral leg (closed circles) and contralateral leg (open circles). Each symbol is the mean iEMG for seven continuous gait cycle intervals. Baseline (horizontal black line) is the aggregate mean iEMG of both legs (ipsilateral and contralateral) soleus muscles in the control condition during the entire 15 min walking period.

The magnitude of integrated EMG of subjects' tibialis anterior and soleus muscles during continuous stepping revealed notable differences between ipsilateral and contralateral legs and between conditions during the 15 min walking period. Walking in the stop condition, the ipsilateral soleus muscle elicited an immediate decrease in iEMG below baseline and a continued gradual decline through the end of the walking period. Conversely, the magnitude of iEMG of the contralateral soleus muscle in the stop condition elicited an immediate increase above baseline, followed by a gradual return to baseline by the end of the walking period. In the free condition, the magnitude of iEMG of subjects' ipsilateral soleus muscle remained at baseline for the first minute followed by a gradual decline below baseline through the remainder of the 15 min walking period (Figure 3). The magnitude of subjects' iEMG of the contralateral soleus muscle in the free condition elicited an immediate increase above baseline followed by a return to baseline at the fourth minute of walking and remained at baseline for the remainder of the walking period (Figure 3). In the control condition, subjects' iEMG of the ipsilateral and contralateral soleus muscles exhibited an immediate increase above baseline followed by a return to baseline by the fourth minute, which persisted at or near the baseline for both legs for the remainder of the walking period (Figure 3).

**Figure 4:**
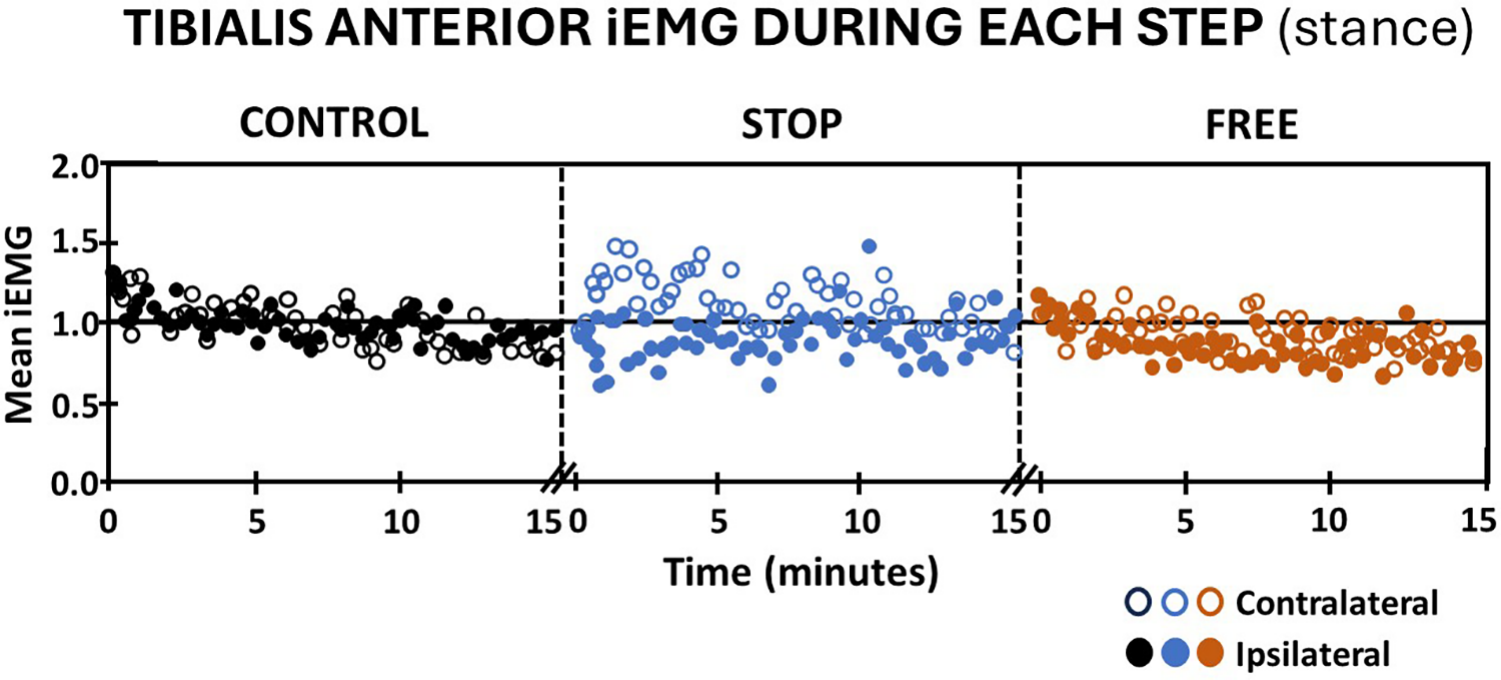
Tibialis anterior muscle activation (mean iEMG) during each step (stance phase) in the control (black), stop (blue), and free (brown) conditions during walking for all subjects (*n* = 14). Note the ipsilateral leg (closed circles) and contralateral leg (open circles). Each symbol represents the mean normalized iEMG for seven continuous gait cycle intervals. Baseline (horizontal black line) is the aggregate mean iEMG of both legs (ipsilateral and contralateral) soleus muscles in the control condition during the entire 15 min walking period.

Because the tibialis anterior muscle elicited activation in stance and swing, iEMG outputs were evaluated independently in the stance and swing phases of gait. Walking in the stop condition during the stance phase elicited an immediate decrease in subjects' iEMG of the ipsilateral tibialis anterior muscle followed by a pattern of variable increase above baseline and decrease below baseline, which persisted to the completion of the 15 min walking period. Conversely, subjects' iEMG of the contralateral tibialis anterior during the stance phase exhibited an immediate increase above baseline during the first 5 min of walking followed by a pattern of variable increase above and decrease below baseline, which persisted during the final 10 min of walking in the stop condition (Figure 4). The free condition elicited an immediate increase in subjects' iEMG of the ipsilateral tibialis anterior during the stance phase followed by a gradual decline below baseline. Walking in the free condition during the stance phase, subjects' iEMG of the ipsilateral tibialis anterior exhibited an immediate increase above baseline followed by a gradual decline below baseline, which persisted to the 15th minute of walking. In the free condition during the stance phase, subjects' contralateral tibialis anterior muscle iEMG exhibited an immediate yet modest increase in activation above baseline followed by a variable pattern of decrease below baseline and increase above baseline (Figure 4). In the control condition, subjects' iEMG of ipsilateral and contralateral tibialis anterior muscle during the stance phase exhibited an immediate increase followed by a gradual decline to baseline by the 15th minute (Figure 4).

**Figure 5:**
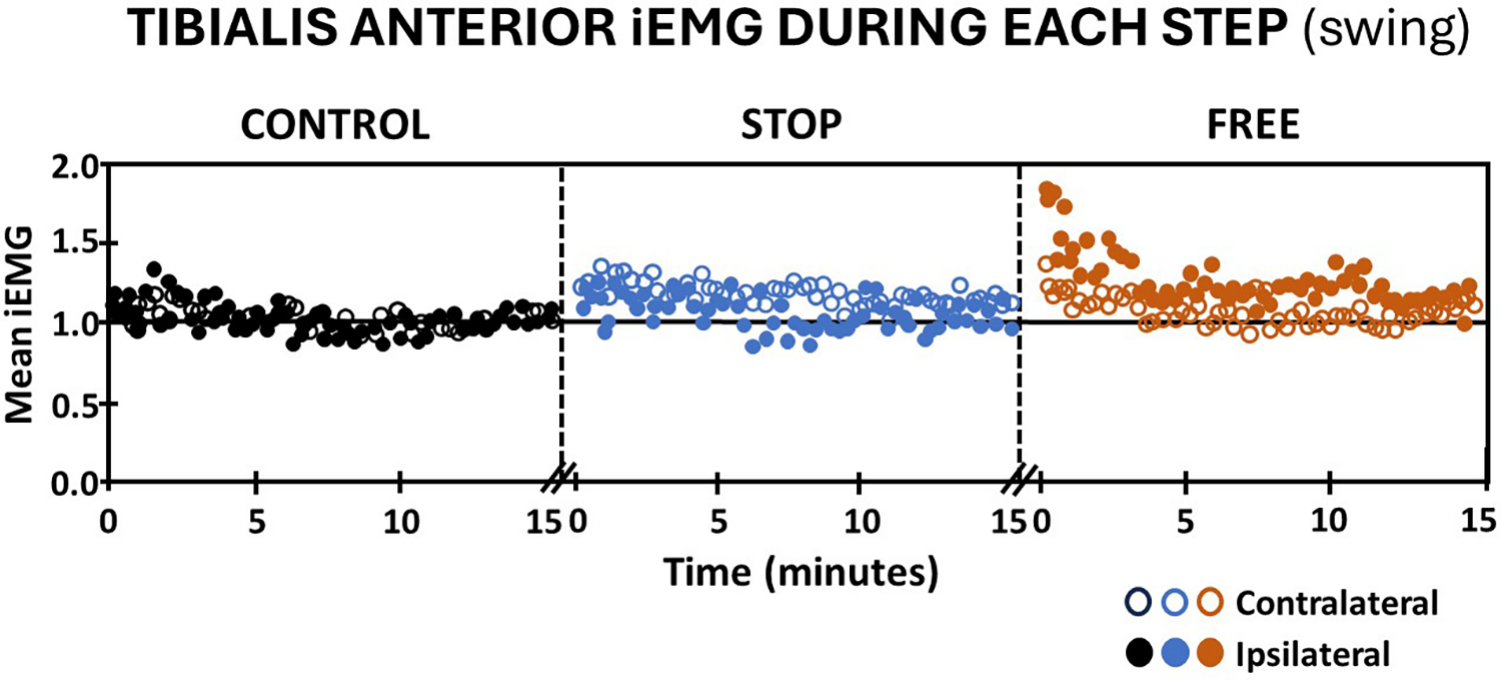
Tibialis anterior muscle activation (mean iEMG) during each step (swing phase) in the control (black), stop (blue), and free (brown) conditions during walking for all subjects (*n* = 14). Note the ipsilateral leg (closed circles) and contralateral leg (open circles). Each symbol represents the mean normalized iEMG for seven continuous gait cycle intervals. Baseline (horizontal black line) is the aggregate mean iEMG of both legs (ipsilateral and contralateral) tibialis anterior muscles in the control condition during the entire 15 min walking period.

Walking in the stop condition during the swing phase, subjects' iEMG of the ipsilateral tibialis anterior elicited an immediate increase followed by a gradual return to baseline, whereas the contralateral tibialis anterior in the stop condition, elicited an immediate and sustained increase in iEMG above baseline (Figure 5). In the free condition, subjects' iEMG of the ipsilateral tibialis anterior exhibited an immediate and substantial increase followed by a gradual decline that remained above baseline throughout the entire 15 min of walking. On the contralateral leg in the free condition, there was an immediate increase in iEMG of the tibialis anterior during swing followed by a return to baseline by the 15th minute of walking. Subjects' iEMG of ipsilateral and contralateral tibialis anterior muscle in the control condition during the swing phase of gait exhibited an immediate increase above baseline followed by a gradual return to at or near baseline in each leg (Figure 5).

#### 3.2.2 Integrated EMG of soleus and tibialis anterior muscles during final 5 min of walking

To further quantify neuromuscular adaptation during walking, the subjects' mean integrated EMG of tibialis anterior and soleus muscles in the stop and free conditions were calculated relative to the control condition during the final 5 min. The final 5 min was selected for analysis because subjects achieved a steady state of iEMG and ankle ROM during this period compared to the prior 10 min of walking.

**Figure 6:**
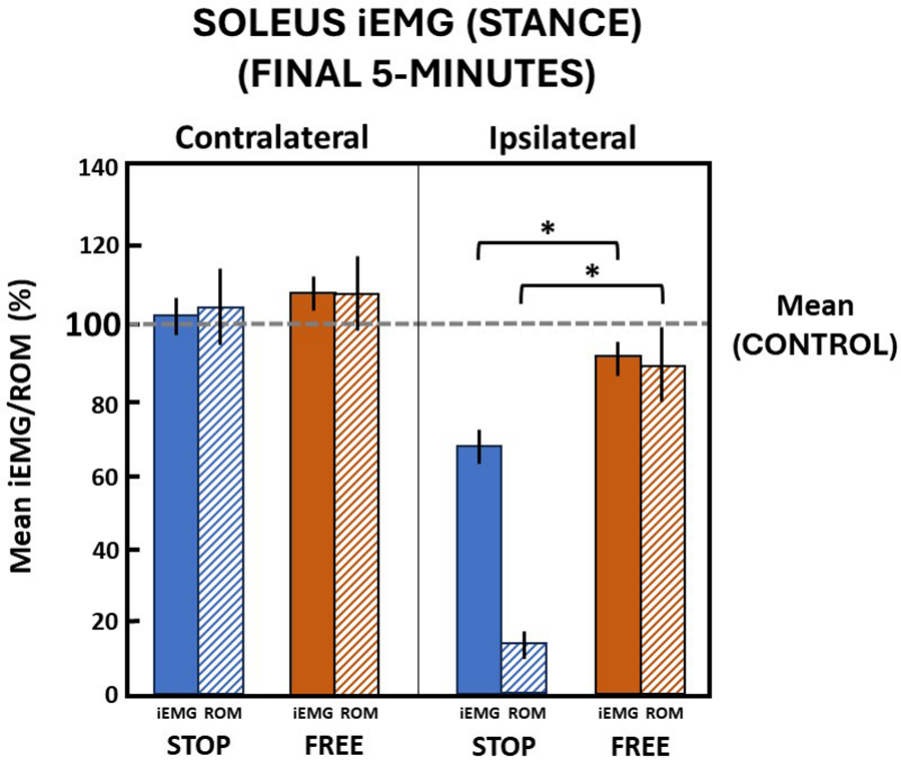
Soleus muscle activation (mean iEMG) in the final 5 min during the stance phase. The horizontal dashed line is the aggregate mean of both legs’ ROM and iEMG expressed as 100% in the control condition. All values relative to the control condition (%). * indicates significance (*p *< 0.05). Ankle range of motion (ROM) and iEMG (%) in the stop (blue) and free (brown) conditions in the ipsilateral (solid) and contralateral (diagonally hatched) legs of all subjects (*n* = 14) during the last 5 min.

During the final 5 min in the stop condition when ankle motion was constrained to mean (standard deviation) 13.1 (2.8)% of the total ROM, subjects' ipsilateral soleus muscle iEMG mean (standard deviation) declined to 67.9 (8.9)% relative to the control condition during the stance phase of gait (Figure 6). In the free condition, when ankle motion was 90.1 (20.1)% of ROM, the ipsilateral soleus muscle iEMG declined to 88.4 (9.2)% relative to the control condition. The difference between ipsilateral soleus iEMG in the stop condition compared to the free condition was significant (*p *= 0.000). On the contralateral leg in the stop condition, the ankle ROM was modestly increased to 104.5 (19.3)% and iEMG was increased to 102.1 (9.4)% in the control condition. In the free condition on the contralateral leg, ankle motion also similarly increased to 107.9 (21.4)% and iEMG increased to 108.1 (8.9)% in the control condition, respectively. There was no difference (*p *> 0.05) in ankle motion and no difference (*p *> 0.05) in iEMG of the soleus muscle between the stop and free conditions on the contralateral leg (Figure 6).

**Figure 7:**
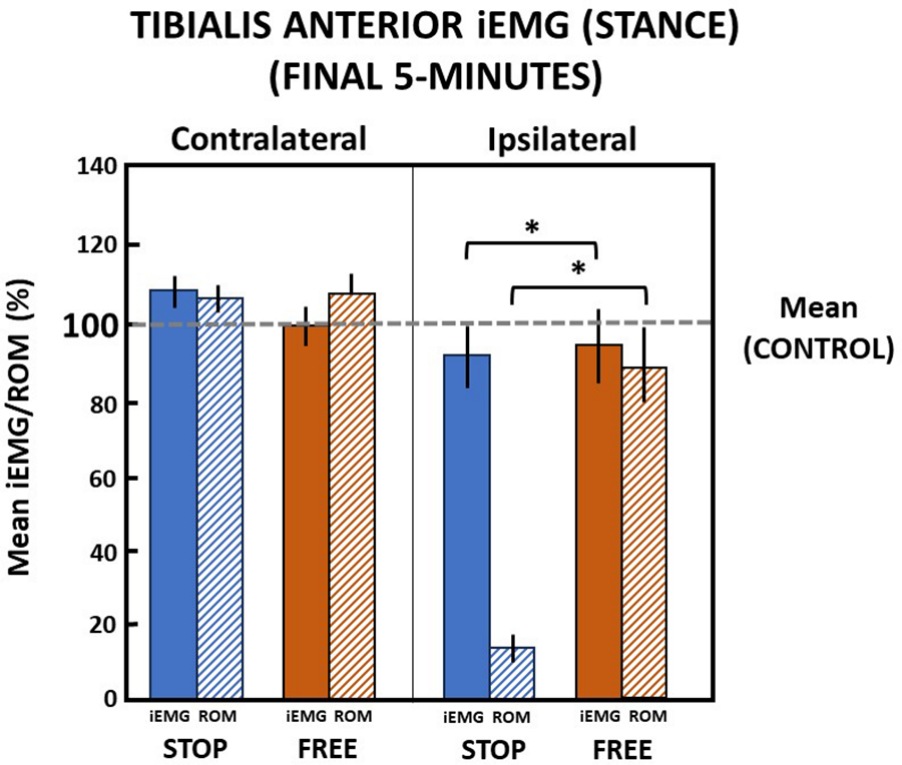
Tibialis anterior muscle (mean iEMG) during the stance phase in the final 5 min. The horizontal dashed line is the aggregate mean of both legs’ ROM and iEMG expressed as 100% in the control condition. All values relative to the control condition (%). *indicates significance (*p *< 0.05). Percent of ankle range of motion (ROM) and percent of iEMG in the stop (blue) and free (brown) conditions in the ipsilateral (solid) and contralateral (diagonally hatched) legs of all subjects (*n* = 14) during the last 5 min.

In the final 5 min in the stop condition when ankle motion was constrained to 13.1 (2.8)% of the ROM, subjects' ipsilateral tibialis anterior muscle iEMG declined to 88.8 (15.4)% relative to the control condition during the stance phase of gait (Figure 7). In the free condition, when ankle motion was 90.1 (20.1)% of the ROM, the ipsilateral tibialis anterior muscle iEMG modestly declined to 95.5 (18.6)% relative to the control condition. Despite a significant (*p *= 0.000) difference between ankle motion in stop and free conditions, there was no difference (*p *> 0.05) in the ipsilateral tibialis anterior muscle iEMG between the stop and free conditions.

On the contralateral leg in the stop condition, the ankle ROM was modestly increased to 106.8 (7.5)% and iEMG of the tibialis anterior was increased to 109.1 (8.5)% of the control condition during the stance phase. In the free condition on the contralateral leg during the stance phase, ankle motion increased to 108.7 (10.1)% and iEMG declined to 99.2 (10.3)% in the control condition. There was no difference (*p *> 0.05) in ankle motion and no difference (*p *> 0.05) between iEMG of the tibialis anterior muscle in the stop and free conditions on the contralateral leg during the stance phase (Figure 7).

**Figure 8:**
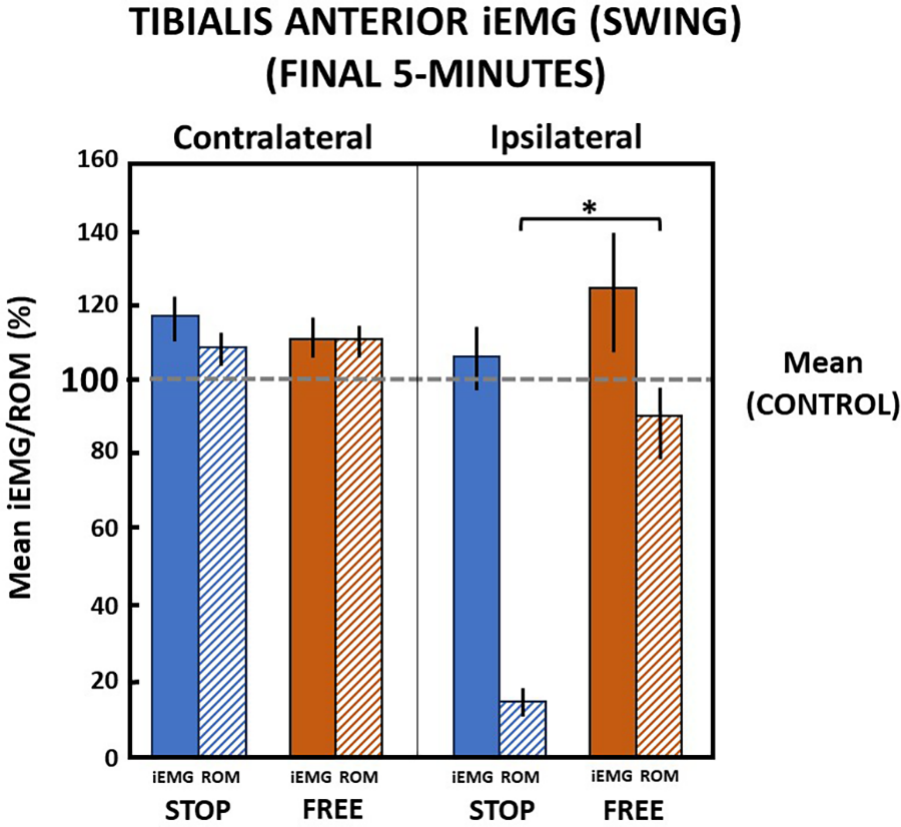
Tibialis anterior muscle (mean iEMG) during swing phase in the final 5 min). The horizontal dashed line is the aggregate mean of both legs’ ROM and iEMG expressed as 100% in the control condition. All values relative to the control condition (%). *indicates significance (*p *< 0.05). Percent of ankle range of motion (ROM) and percent of iEMG in the stop (blue) and free (brown) conditions in the ipsilateral (solid) and contralateral (diagonally hatched) legs of all subjects (*n* = 14) during the last 5 min.

Walking in the final 5 min in the stop condition when ankle motion was constrained to 13.1 (2.8)% of the ROM, subjects' ipsilateral tibialis anterior muscle iEMG increased to 106.6 (17.1)% relative to the control condition during the swing phase of gait (Figure 8). In the free condition, when ankle motion was 90.1 (20.1)% of the ROM, the ipsilateral tibialis anterior muscle iEMG notably increased to 123.7 (32.4)% relative to the control condition. Despite a significant (*p *= 0.000) difference between ankle motion in stop and free conditions, there was no difference (*p *> 0.05) in the ipsilateral tibialis anterior muscle iEMG between stop and free conditions during the swing phase (Figure 8).

On the contralateral leg in the stop condition, the ankle ROM moderately increased to 110.2 (9.9)% and iEMG of the tibialis anterior moderately increased to 118.5 (12.5)% of the control condition during the swing phase. In the free condition on the contralateral leg during the swing phase, ankle motion similarly increased to 111.9 (9.5)% and iEMG increased to 111.8 (11.2)% in the control condition. There was no difference (*p *> 0.05) in ankle motion and no difference (*p *> 0.05) between iEMG of the tibialis anterior muscle in stop and free conditions on the contralateral leg during the swing phase (Figure 8).

## 4 Discussion

Traditionally most orthotic interventions are founded on the mechanics of body segment and joint motion control with little or no consideration of the consequential sensorimotor response to a particular orthotic intervention. This narrow clinical perspective is due, in part, to our limited knowledge of the neuromuscular mechanisms associated with the use of orthoses and footwear. Only three studies investigated the lower limb muscle EMG of healthy subjects using an AFO (28, 29, 32). While these investigations evaluated healthy subjects, the methods were substantially different such that they employed non-standardized footwear, substantially different AFO designs providing variable ankle motion control, and different methods of EMG digital signal processing and analysis. Given the numerous differences, comparisons of these studies to findings in our investigation are difficult to interpret.

Data from our investigation supports an emergent theory that when ankle joint motion is constrained by the use of a lower limb orthosis during walking, skeletal muscle activation of uni-articular muscles acting on the constrained ankle joint is altered compared to unconstrained walking. A summary of preliminary findings including a description of the characteristics of an orthosis-induced neuromotor response mechanism due to constraint of ankle motion is described.

The constraint of ankle joint motion of subjects walking in the exAFO-FC altered the activation of the soleus and tibialis anterior muscles. During the stance phase of walking in the AFO and footwear conditions, the ipsilateral soleus muscle iEMG progressively declined linearly over continuous steps, culminating in a 32.1% reduction compared to the control condition in the final 5 min of a 15 min protocol. The ipsilateral tibialis anterior muscle iEMG declined 11.2% in the final 5 min of walking, compared to the control condition. Unlike the linear decline over continued steps observed by the ipsilateral soleus muscle, the iEMG output of the ipsilateral tibialis anterior muscle was highly variable in the same respective test parameters. During the swing phase walking during maximal constraint of ankle joint motion in the AFO, the ipsilateral tibialis anterior muscle again exhibited a high variation in iEMG during continuous stepping. This culminated in a 6.6% increase in iEMG compared to the control condition during the final 5 min of the 15 min period of walking. Hence, during the swing phase, the tibialis anterior muscle demonstrated an increase in iEMG in the final 5 min compared to declines in iEMG exhibited by the soleus and tibialis anterior muscles during the stance phase of gait.

These findings may not have been observed previously because the study described herein used a novel AFO specifically designed to optimize ankle joint constraint of motion. Additionally, the experimental protocol employed continuous sampling of muscle EMG during walking, which was well-suited to study neuromuscular behavior to the constraint of motion. Further discussion of the soleus and tibialis anterior muscle response coupled with the specialized ankle motion constraint design and performance of the experimental AFO and integrated footwear help explain the clinical implications ascertained from the preliminary results in this investigation.

Our investigation leveraged an AFO that delivered near-total ankle constraint of plantarflexion and dorsiflexion motion during gait. An experimental AFO-footwear combination was developed, rigorously tested, and validated to restrict ankle movement to less than 4° of dorsiflexion and plantarflexion motion (3). The AFO and integrated footwear were assessed in two performance studies: (a) a quasistatic loading study using cadaveric limbs to quantify the motion control capability of the experimental AFO and (b) a gait study involving human subjects to quantify the combined effectiveness of the exAFO and integrated footwear for motion control and preservation of rollover. These studies provided supportive evidence that the footwear design features contribute to maintaining rollover and minimized interruption of forward progression, despite the restriction of ankle motion provided by the exAFO-FC (3).

Our approach to collecting and analyzing muscle EMG was based on the premise that EMG samples the muscle's electrical activity and is representative of muscle action potentials. Hence, sampling and analysis of muscle EMG can provide insights into the neural control system and its influence on neuromuscular output. Based on this premise, we applied a twofold method of examining muscle EMG. First, we characterized the neuromuscular response of the soleus and tibialis anterior muscles to the motion control conditions (control, stop, free) by collecting and analyzing EMG during continuous stepping in a 15 min walking protocol. Second, we quantified the muscle EMG and collapsed these data in the final 5 min interval of walking as a representation of the adaptation of each muscle to the experimental conditions.

The twofold method of examining the EMG of soleus and tibialis anterior muscles during the walking protocol yielded data sets that enabled the interpretation of their neuromuscular behavior and adaptation to the constraint of motion. Key findings from this analysis are that the ipsilateral soleus and tibialis anterior exhibit different patterns of output to the constraint of motion. During the stance phase, the soleus muscle exhibits a linear and non-variable decline during continuous stepping in the AFO and footwear whereas the tibialis anterior muscle exhibits a highly variable decline. The decline in soleus muscle activation is nearly three times the magnitude of the decline in tibialis anterior muscle activation. In the swing phase, the ipsilateral tibialis anterior muscle demonstrates a variable increase (rather than decrease) in activation during the 15 min walking period. This suggests that despite similarities as one-joint muscles that typically engage in eccentric lengthening contraction during the stance phase, the soleus and tibialis anterior muscle iEMG during constraint of motion is altered in a way that regulates the magnitude of activation differently. Furthermore, the phase of gait during constraint of motion may influence the direction of neuromuscular output during constraint of motion (e.g., stance phase decline and swing increase). Finally, the magnitude of the constraint of motion appears to relate to the magnitude of the decline in muscle activation. This is supported by iEMG of subjects walking in the free condition where ankle motion was minimally constrained and was similar to the control condition. During minimal constraint of ankle motion by the AFO in the free condition, the ipsilateral soleus and tibialis anterior muscles exhibited only modest decreases in iEMG during the stance phase of gait. Conversely, walking in the free condition during the swing phase, the ipsilateral tibialis anterior muscle exhibited a substantial increase in activation. This may be due in part to the inertial effects of the AFO evoking increased activation of the muscle to dorsiflex the ankle and lift the mass of the foot and AFO to ensure foot clearance from the ground.

A plausible explanation and perhaps a limitation of the study may relate to the mass of the exAFO-FC. The majority of the mass in the experimental AFO and footwear system was due to the adjustable ankle motion linear bearing component located at the shank. This was a favorable location because it concentrated the mass in a more proximal position on the leg (as opposed to the ankle). We conducted a pilot study of 14 healthy subjects walking in the exAFO and in a control (no AFO) condition to examine the potential for inertial effects. We found no differences (*p *> 0.05) in subjects' heart rate, perceived exertion, preferred overground walking speed, and cadence, yet there were modest but significant (*p *< 0.05) differences in stance duration (4%) and swing duration (6%). A portion of these findings appear in a prior study (3).

Other investigators studied inertial effects by incrementally varying the location of mass added to the leg of healthy subjects and found steadily increasing metabolic costs with more distal placement due to changes in the moment of inertia (53). Skinner and Barrack reported the addition of 1.82 kg mass of a single leg at the ankle of healthy subjects elicited a modest (7%) increase in oxygen consumption but no difference (*p *> 0.05) in velocity, cadence, stride length, gait cycle, and double-limb support time (54). They did report alterations in single limb support time (decreased) and swing phase (increased) compared to the control (no added weight) condition. Based on the comparison of these findings to our study, the concentration of mass in the exAFO-FC at the shank likely minimizes inertial effects and their influence on the gait of subjects. This is supported by subjects demonstrating modest differences in stance (4%) and swing (6%) phase duration and no differences in walking speed, cadence, heart rate perceived exertion, and preferred speed in exAFO-FC compared to the control (no AFO) condition.

A likely explanation for the decline in EMG activity during constraint of joint motion is that the orthosis provides external biomechanical stabilization of the ankle joint, which (without the orthosis) is normally provided through muscular action. During orthosis use, the neural control system responds in a way to minimize the effort needed when the ankle joint can be stabilized without neuromuscular activity. The magnitude of the decline is somewhat proportional to the magnitude of the motion constraint. The greater the constraint of ankle motion (e.g., stop condition) evokes a greater decline in neuromuscular activity, whereas the lower the level of ankle constraint of motion (e.g., free condition) evokes a lower decline in neuromuscular activity. This may not be surprising but in this specific clinically relevant context, the physiological adaptation in response to mechanical constraint results in reduced muscle activity.

Key findings of the contralateral leg iEMG align with findings from the ipsilateral leg iEMG and further support the proposed adaptation to the constraint of motion using the experimental AFO and integrated footwear. During maximal constraint of motion of the ipsilateral leg, iEMG of the contralateral soleus and tibialis anterior muscles during the stance phase exhibited an increase above baseline during continuous stepping that declined near baseline at the end of the 15 min walking period. Similar to the ipsilateral leg, the contralateral soleus muscle exhibited a linear pattern of decline, and the contralateral tibialis anterior exhibited a variable pattern of decline respectively. During the swing phase, the contralateral tibialis anterior iEMG followed a similar pattern as during the stance phase on the contralateral side. Hence, an adaptive change in iEMG output during use of the AFO and footwear occurred in the contralateral soleus and tibialis anterior muscles in a similar fashion to the ipsilateral leg. To further complement these EMG results, the ankle joint motion and moments were no different (*p *> 0.05) in the contralateral leg when subjects walked in the experimental conditions [i.e., AFO in maximal ankle constraint (stop condition) and free ankle motion (free condition)] compared to the control (no AFO) condition (3). This supports that the contralateral leg did not experience notable compensatory movements despite the orthotic constraint of ankle motion on the ipsilateral leg.

The implications of these findings are that unilateral constraint of lower limb motion using an AFO and footwear during walking influences the neuromuscular behavior of skeletal muscles on both lower limbs. Generally, the pattern of behavior is a decline in neuromuscular activity on the ipsilateral constrained leg and an increase in neuromuscular activity on the contralateral leg.

## 5 Conclusion

When an orthosis constrains ankle joint movement during walking, an adaptative neuromotor response mechanism will alter neuromuscular output with progressive stepping (e.g., 15 min of walking) that changes iEMG activity compared to an unconstrained control. Clinicians need to be cognizant of this adaptive response period when planning treatments, particularly in users who do not have a neuromotor deficit. Additionally, the motion blocking and footwear features incorporated into an orthosis system are likely critical factors to the effectual neuromotor response of the user. Further study of these parameters in clinical populations is needed to confirm the findings in this study of healthy subjects.

## Data Availability

The raw data supporting the conclusions of this article will be made available by the authors, without undue reservation.
